# Cross-sectional study of ethnic differences in physical fitness among children of South Asian, black African–Caribbean and white European origin: the Child Heart and Health Study in England (CHASE)

**DOI:** 10.1136/bmjopen-2016-011131

**Published:** 2016-06-20

**Authors:** C M Nightingale, A S Donin, S R Kerry, C G Owen, A R Rudnicka, S Brage, K L Westgate, U Ekelund, D G Cook, P H Whincup

**Affiliations:** 1 Population Health Research Institute, St George's, University of London, London, UK; 2 Centre for Primary Care and Public Health, Queen Mary, University of London, London, UK; 3 MRC Epidemiology Unit, University of Cambridge School of Clinical Medicine, Institute of Metabolic Science, Cambridge, UK; 4 Department of Sport Medicine, Norwegian School of Sport Sciences, Oslo, Norway

**Keywords:** Physical fitness, Children, Ethnicity

## Abstract

**Objective:**

Little is known about levels of physical fitness in children from different ethnic groups in the UK. We therefore studied physical fitness in UK children (aged 9–10 years) of South Asian, black African–Caribbean and white European origin.

**Design:**

Cross-sectional study.

**Setting:**

Primary schools in the UK.

**Participants:**

1625 children (aged 9–10 years) of South Asian, black African–Caribbean and white European origin in the UK studied between 2006 and 2007.

**Outcome measures:**

A step test assessed submaximal physical fitness from which estimated VO_2 max_ was derived. Ethnic differences in estimated VO_2 max_ were estimated using multilevel linear regression allowing for clustering at school level and adjusting for age, sex and month as fixed effects.

**Results:**

The study response rate was 63%. In adjusted analyses, boys had higher levels of estimated VO_2 max_ than girls (mean difference 3.06 mL O_2_/min/kg, 95% CI 2.66 to 3.47, p<0.0001). Levels of estimated VO_2 max_ were lower in South Asians than those in white Europeans (mean difference −0.79 mL O_2_/min/kg, 95% CI −1.41 to −0.18, p=0.01); levels of estimated VO_2 max_ in black African–Caribbeans were higher than those in white Europeans (mean difference 0.60 mL O_2_/min/kg, 95% CI 0.02 to 1.17, p=0.04); these patterns were similar in boys and girls. The lower estimated VO_2 max_ in South Asians, compared to white Europeans, was consistent among Indian, Pakistani and Bangladeshi children and was attenuated by 78% after adjustment for objectively measured physical activity (average daily steps).

**Conclusions:**

South Asian children have lower levels of physical fitness than white Europeans and black African–Caribbeans in the UK. This ethnic difference in physical fitness is at least partly explained by ethnic differences in physical activity.

**Strengths and limitations of this study**

- The submaximal fitness test used to predict VO_2 max_ has previously been employed in a nationally representative sample of the English population.
- Ethnic comparisons carried out on a within-school basis to limit confounding with balanced representation of South Asians, black African–Caribbeans and white Europeans.
- Objective accelerometer-based levels of physical activity levels were made using a validated method and bioelectrical impedance was used to provide a valid measurement of adiposity in this multi-ethnic population.
- The response rate of 63% was modest and estimated VO_2 max_ was obtained for 75% of the sample who completed the step test; however, characteristics of participants with and without physical fitness data were similar.

## Introduction

In the UK and in other western European countries, there are marked ethnic differences in chronic disease risks. British South Asian adults have increased risks of developing type 2 diabetes, coronary heart disease and stroke compared to white Europeans in the UK.^1^ ^2^ British black African–Caribbeans, in contrast, have increased risks of type 2 diabetes and stroke compared to white Europeans but lower risks of coronary heart disease.^1^ ^2^ Recent evidence suggests that these ethnic differences in disease risks have their origins in childhood, with increased levels of insulin resistance, circulating glucose and body fatness in South Asian children^3^ ^4^ and (to a lesser extent) black African–Caribbean children compared to white Europeans.^4^ Marked ethnic differences in physical activity levels have been reported in the UK in adults^5^ and children,^6^ with South Asians in particular having lower physical activity levels than white Europeans. However, little is known about the extent of ethnic differences in physical fitness, a determinant of cardiovascular and metabolic risk in adults^7–9^ and children.^10^ An earlier report based on a small number of ethnic minority children studied in the National Study of Health and Growth raised the possibility that such differences could be substantial and that they might well be explained by ethnic differences in physical activity.^11^ The importance of independent assessment of physical fitness is emphasised by the results of studies, showing that physical fitness and physical activity are independently associated with metabolic risk^10^ and that physical fitness may be a stronger risk factor than physical activity for coronary heart disease^12^ and all-cause mortality.^13^ We have therefore examined ethnic differences in physical fitness in a study of British school children of South Asian, black African–Caribbean and white European origin, presenting data on the major ethnic groups and subcategories. We have particularly examined the extent to which ethnic differences in physical fitness can be explained by objectively measured physical activity (overall intensity and moderate to vigorous intensity), an important determinant of physical fitness,^10^ ^14^ and by adiposity, which is inversely associated with physical fitness.^10^

## Research design and methods

### Study design

The Child Heart and Health Study in England (CHASE) was a cross-sectional investigation of the health (particularly cardiovascular and metabolic health) of primary school children aged 9–10 years of white European, South Asian and black African–Caribbean origin in the UK carried out between October 2004 and February 2007; full details have been published elsewhere.^4^ ^6^ ^15^ Ethical approval was obtained from the relevant multicentre research ethics committee and written, informed parental consent was obtained for all participating children. The study was based in 200 state primary schools in London, Birmingham and Leicester, half with a high prevalence of UK South Asian children (stratified by Indian, Pakistani and Bangladeshi origin) and other half with a high prevalence of UK black African–Caribbean children (stratified by black African and black Caribbean origin). Schools were drawn at random from the stratified sampling frame; schools which declined to participate were replaced by another similar school within the sampling frame. This report is based on the final phase of the study (81 schools studied between January 2006 and February 2007) in which additional assessments of physical fitness and physical activity were made.

### Survey measurements

A single survey team of three trained Research Nurses and two Research Assistants carried out all assessments; each observer measured approximately one-third of children in each ethnic group. Participating children had physical measurements including height and weight, and arm-to-leg bioelectrical impedance, using the Bodystat 1500 bioelectrical impedance monitor (Bodystat, Isle of Man, UK). Fat mass was derived from impedance using ethnic- and gender-specific equations derived for UK children of this age group.^16^ Fat mass index (FMI) [fat mass (kg)/height(m)^5^] was derived to be independent of height (r=−0.02)^16^ and was shown to be a more valid marker of body fatness than body mass index in this study population.^17^

Participants underwent an 8-min step test as described previously.^18^ ^19^ Briefly, participants were fitted with a combined heart rate (HR) and movement sensor (Actiheart, CamNtech, Papworth, UK), then followed an audible prompt instructing them to progressively increase their step frequency, ramping from 15 to 32.5 body lifts/min (rate of change: 2.5 body lifts/min^2^) on a 150 mm high step. The step test was terminated if the participant was unable to maintain the prescribed step frequency, even after verbal encouragement from the investigator. After test termination, 2 min of seated recovery was measured. The combined sensor-recorded ECG and acceleration waveforms (128 and 32 Hz sampling, respectively), were summarised in 15 s epochs. Data were visually reviewed and noisy ECG data were excluded from analysis. Heart beats were detected using a modified Pan-Tompkins peak detection algorithm.^18^ Estimation of VO_2 max_ was done in a similar manner as Health Survey for England 2008.^20^ Briefly, predicted workload was regressed against instantaneous HR (expressed above resting level) and 1-min recovery HR was extracted using quadratic regression against recovery time (first 90 s); these parameters were combined with resting HR and test duration to define the submaximal relationship between HR and workload, which was then extrapolated to predicted maximal HR^21^ to predict maximal work capacity. This was converted to VO_2 max_ by first adding an estimate of resting metabolic rate^22^ and then dividing by the energetic value of oxygen to estimate.^20^

Physical activity was assessed by accelerometry; children were asked to wear an activity monitor (GT1M; ActiGraph LLC, Pensacola, Florida, USA), on their left hip during waking hours (apart from during water-based activities) for 7 days following measurement and then return the instrument to the school. The ActiGraph monitor was worn over the left hip on an elasticised belt. Non-wear time, defined as periods of at least 20 consecutive minutes of zero counts, was excluded and remaining data were summarised into mean daily counts, counts per minute (CPM), steps per day and time spent at moderate to vigorous intensity (moderate–vigorous physical activity, MVPA; ≥2000 CPM counts). All participants with one or more days of valid data were included in the analysis; a valid day being defined as at least 600 min of registered time.

### Ethnicity and socioeconomic status

The ethnicity of the child was defined using parental information on the self-reported ethnicity of both parents where available (63%), or using the parentally defined ethnic origin of the child (36%), or using information on parental and grand-parental place of birth provided by the child, cross-checked with observer assessment of ethnic origin (1%). Children were broadly defined (as previously described^4^) as white European, South Asian, black African–Caribbean and other ethnicity; more detailed ethnic subcategories of South Asians (Indian, Pakistani, Bangladeshi and South Asian other) and black African–Caribbeans (black African, black Caribbean and black other) were also used in analyses. Parents and children provided information on parental occupation, which was coded using the National Statistics-Socioeconomic Classification (NS-SEC) as previously described.^23^

### Statistical methods

Statistical analyses were carried out using Stata/SE software (Stata/SE V.12 for Windows; StataCorp LP, College Station, Texas, USA). Estimated VO_2 max_ was normally distributed (see online supplementary figure S1). We used a previously published regression calibration method to allow for measurement error in the physical activity variables (counts, CPM, steps and moderate to vigorous activity).^24^ This method allows for within-child variation in physical activity across a variable number of days of recording (between 1 and 7) and by day of the week and provides an unbiased average of counts and CPM for each child. Most children (87%) had 3 or more full days of recorded physical activity data. Restricting the analyses to these children did not materially affect the results.

10.1136/bmjopen-2016-011131.supp1 Supplementary data

Gender differences in estimated VO_2 max_ were assessed using multilevel models adjusted for age, ethnicity and month of the year (fitted as fixed effects) and school fitted as a random effect to take account of clustering of children within schools; all models were fitted using the *xtmixed* command in Stata. Similar models were used to quantify ethnic differences in estimated VO_2 max_ adjusted for age, sex, month and school. An interaction between ethnic group and sex was fitted and likelihood ratio tests were used to examine whether ethnic differences in physical fitness were modified by sex. Associations between estimated VO_2 max_ and physical activity counts, CPM, fat mass index and resting HR were plotted and were quantified using correlation coefficients. To examine whether ethnic differences in estimated VO_2 max_ were explained by ethnic differences in physical activity, activity counts, CPM, steps or time spent in moderate to vigorous activity was fitted as a covariate in the model; fat mass index was fitted as a covariate to examine whether adiposity accounted for ethnic differences in estimated VO_2 max_.

## Results

Of 3571 children invited to participate in this phase of the study, 2236 (63%) took part in the physical fitness test. Response rates were similar among South Asians and white Europeans and other ethnic groups (67%, 65% and 63%, respectively) but slightly lower among black African–Caribbeans (59%); response rates were higher in girls than boys (66% and 59%, respectively). Among 2236 children who took part in the test, 73 participants were removed from the analysis due to not maintaining prescribed step frequency during the test and 538 participants did not have adequate HR data. Estimated VO_2 max_ values were therefore derived for 1625 participants; the proportion of those with valid physical fitness data was similar in boys and girls and unrelated to ethnicity, socioeconomic position or physical characteristics. The children with estimated VO_2 max_ values included similar numbers of boys and girls (825 and 800, respectively) and similar numbers of children of white European, South Asian, black African–Caribbean and other ethnicity (424, 407, 413 and 381, respectively). Of these children, 1215 also had objective measures of physical activity.

Unadjusted means and SDs for estimated VO_2 max_ are shown by gender and ethnic group in online supplementary table S1; adjusted mean levels and gender differences in VO_2 max_ are shown in table 1. The overall level of estimated VO_2 max_ in the study population was 39.4 mL O_2_/min/kg (95% reference range 30.6, 48.2). Girls had markedly lower levels of estimated VO_2 max_ than boys; on average, the level of estimated VO_2 max_ in girls was 3.06 mL O_2_/min/kg lower (95% CI 2.66 to 3.47) than that in boys. This gender difference was apparent in all individual ethnic groups and there was no strong evidence of an interaction between ethnic group and sex (p=0.33).

**Table 1:** Adjusted means for estimated VO_2 max_ (mL O_2_/min/kg) by sex and ethnic group

		Mean estimated VO_2 max_ (95% CI)							
Ethnic group or subgroup	n	Boys (n=825)		Girls (n=800)		p (sex difference)	All		p (ethnicity)*
All children	1625	40.9	(40.5 to 41.3)	37.8	(37.4 to 38.2)	<0.0001	39.4	(39.0 to 39.7)	
White European	424	40.7	(40.1 to 41.3)	38.1	(37.5 to 38.8)	<0.0001	39.4	(39.0 to 39.9)	
South Asian	407	40.0	(39.4 to 40.7)	37.2	(36.5 to 37.8)	<0.0001	38.6	(38.1 to 39.2)	0.13
Indian	111	39.6	(38.5 to 40.7)	36.2	(35.0 to 37.5)	<0.0001	37.9	(37.1 to 38.8)	
Pakistani	147	39.9	(38.9 to 40.9)	37.2	(36.1 to 38.2)	<0.0001	38.6	(37.8 to 39.3)	
Bangladeshi	121	40.6	(39.4 to 41.8)	37.5	(36.5 to 38.5)	<0.0001	39.1	(38.2 to 39.9)	
Black African–Caribbean	413	41.8	(41.1 to 42.4)	38.3	(37.6 to 38.9)	<0.0001	40.0	(39.6 to 40.5)	0.07
Black African	230	42.4	(41.6 to 43.2)	38.4	(37.6 to 39.2)	<0.0001	40.4	(39.8 to 41.0)	
Black Caribbean	148	40.8	(39.9 to 41.8)	38.3	(37.3 to 39.3)	<0.001	39.6	(38.9 to 40.3)	
Other	381	41.0	(40.4 to 41.7)	37.7	(37.0 to 38.3)	<0.0001	39.4	(38.9 to 39.9)	

Adjusted for age quartiles, month, sex, ethnic group, an interaction between ethnic group and sex (except for analysis of all ethnic groups combined) and school (random effect).

South Asian other and black other subgroups are not included in the table; therefore, the numbers in the subgroups do not add up to the main ethnic group totals for South Asians and black African–Caribbeans.

*p Value for heterogeneity between ethnic subgroups within broader ethnic groups, that is, South Asian and black African–Caribbean.

Mean levels of estimated VO_2 max_ for white European, South Asian, black African–Caribbean and ‘other’ ethnic groups are presented in table 1 and adjusted differences compared to white Europeans are summarised in table 2. South Asian children had lower levels and black African–Caribbeans had higher levels of estimated VO_2 max_ than white Europeans, whereas levels of estimated VO_2 max_ in ‘other ethnicity’ were similar to those in white Europeans. Within ethnic minority groups, there was no strong evidence of heterogeneity between Indian, Pakistani and Bangladeshi children, all of whom had lower estimated VO_2 max_ values than white Europeans. Estimated VO_2 max_ levels were higher in black African–Caribbean children, with the difference apparently concentrated in boys and black African children; the test for heterogeneity between black Africans and Caribbeans was of borderline statistical significance (p=0.07).

**Table 2:** Ethnic differences in estimated VO_2__max_ (mL O_2_/min/kg): overall and by sex

	Difference in estimated VO_2 max_ (95% CI), p value								
Compared to white Europeans	Boys (n=825)			Girls (n=800)			All		
South Asian	−0.69	(−1.51 to 0.14)	0.10	−0.95	(−1.79 to −0.11)	0.03	−0.79	(−1.41 to −0.18)	0.01
Indian	−1.16	(−2.36 to 0.03)	0.06	−1.93	(−3.29 to −0.58)	0.01	−1.52	(−2.45 to −0.59)	0.001
Pakistani	−0.85	(−1.98 to 0.29)	0.14	−0.98	(−2.15 to 0.19)	0.10	−0.90	(−1.75 to −0.05)	0.04
Bangladeshi	−0.15	(−1.48 to 1.19)	0.83	−0.67	(−1.83 to 0.49)	0.26	−0.38	(−1.29 to 0.53)	0.42
Black African–Caribbean	1.03	(0.25 to 1.82)	0.01	0.13	(−0.69 to 0.94)	0.76	0.60	(0.02 to 1.17)	0.04
Black African	1.64	(0.70 to 2.58)	<0.001	0.24	(−0.71 to 1.19)	0.62	0.95	(0.27 to 1.62)	0.01
Black Caribbean	0.10	(−0.99 to 1.18)	0.86	0.17	(−0.96 to 1.29)	0.77	0.14	(−0.65 to 0.93)	0.72
Other	0.31	(−0.50 to 1.11)	0.46	−0.48	(−1.31 to 0.34)	0.25	−0.07	(−0.65 to 0.51)	0.80

Adjusted for sex, age quartiles, month, ethnic group, an interaction between ethnic group and sex (except for analysis of all children combined) and school (random effect).

Estimated VO_2 max_ was positively correlated with physical activity counts, CPM, steps and time spent in MVPA; correlation coefficients (r) were 0.40, 0.35, 0.34 and 0.37 respectively. Estimated VO_2 max_ was inversely correlated with resting HR (r=−0.66) and fat mass index (r=−0.37). As reported previously^6^ and shown in online supplementary table S2, physical activity levels (including counts, CPM, steps and MVPA) were lower in South Asians compared to white Europeans; black African–Caribbeans had similar levels of physical activity (CPM and MVPA), though mean levels of counts were higher and mean levels of steps were lower. Adiposity levels (particularly fat mass index) were previously reported in the larger CHASE study to be higher among South Asians compared to white Europeans and similar among black African–Caribbeans compared to white Europeans.^17^ In this smaller subset of the CHASE study, fat mass index was higher in South Asians, though the difference was not statistically significant, and lower in black African–Caribbeans compared to white Europeans.

The effect of adjustment for physical activity (counts, CPM, steps or MVPA) and adiposity (fat mass index) on the overall ethnic differences in physical fitness are provided in table 3 for the subset of data with measurements of physical activity and adiposity. The higher level of estimated VO_2 max_ in black African–Caribbean children compared to white Europeans was reduced by 25% when adjusting for objectively measured physical activity counts; adjustment for steps increased the difference by 34%. Adjustment for fat mass index reduced the difference in estimated VO_2 max_ by 51% on its own and by 54% in combination with adjustment for physical activity (table 3). The lower level of estimated VO_2 max_ in South Asian children compared to white Europeans was not statistically significantly different in this subset; adjustment for objectively measured physical activity further reduced this difference. For South Asians, adjustment for fat mass index did not have an appreciable effect on the ethnic difference in estimated VO_2 max_ either as a single adjustment or in combination with adjustment for physical activity (table 3).

**Table 3:** Ethnic and sex differences in estimated VO_2__max_ (mL O_2_/min/kg): effect of adjustment for physical activity levels and fat mass index

	Difference in estimated VO_2 max_ (95% CI), p value								
Adjustments (N=1215)	South Asian–white European			Black African–Caribbean–white European			Girls–Boys		
Standard	−0.63	(−1.36 to 0.11)	0.09	0.80	(0.14 to 1.46)	0.02	−3.05	(−3.52 to −2.58)	<0.0001
Standard+counts	−0.34	(−1.05 to 0.37)	0.35	0.60	(−0.04 to 1.24)	0.07	−1.96	(−2.47 to −1.45)	<0.0001
Standard+CPM	−0.19	(−0.91 to 0.53)	0.61	0.81	(0.17 to 1.46)	0.01	−2.21	(−2.71 to −1.71)	<0.0001
Standard+steps	−0.14	(−0.87 to 0.59)	0.71	1.07	(0.42 to 1.73)	0.001	−2.12	(−2.64 to −1.60)	<0.0001
Standard+MVPA	−0.22	(−0.95 to 0.50)	0.54	0.72	(0.08 to 1.37)	0.03	−1.99	(−2.51 to −1.46)	<0.0001
Standard+FMI	−0.57	(−1.25 to 0.12)	0.11	0.39	(−0.24 to 1.01)	0.22	−2.61	(−3.05 to −2.16)	<0.0001
Standard+counts+FMI	−0.36	(−1.03 to 0.31)	0.29	0.29	(−0.32 to 0.90)	0.35	−1.87	(−2.36 to −1.39)	<0.0001
Standard+CPM+FMI	−0.26	(−0.95 to 0.42)	0.45	0.44	(−0.18 to 1.05)	0.16	−2.06	(−2.54 to −1.59)	<0.0001
Standard+steps+FMI	−0.19	(−0.88 to 0.49)	0.58	0.63	(0.01 to 1.24)	0.05	−1.92	(−2.41 to −1.42)	<0.0001
Standard+MVPA+FMI	−0.28	(−0.96 to 0.40)	0.42	0.37	(−0.25 to 0.98)	0.24	−1.88	(−2.38 to −1.38)	<0.0001

Analysis carried out in a subset of 1215 children with objective measurements of physical activity.

Standard adjustment is for sex, age quartiles, ethnicity, month and school (random effect).CPM, counts per minute; FMI, fat mass index; MVPA, moderate to vigorous physical activity.

In similar analyses, the effects of adjustment for physical activity and adiposity on gender differences in estimated VO_2 max_ are given in table 3. For all ethnic groups combined, girls had a markedly lower level of estimated VO_2 max_ compared to boys; this difference was reduced by 36% by adjustment for physical activity counts, by 28% for CPM, by 30% for steps, by 35% for moderate to vigorous activity and by 14% for fat mass index, though the differences were still highly statistically significant. Adjustment for fat mass index in combination with adjustment for counts or CPM did not appreciably reduce the difference further.

In further analyses, estimated VO_2 max_ did not vary appreciably by socioeconomic status (NS-SEC), either overall (p[heterogeneity]=0.08) or within specific ethnic groups; furthermore, adjustment for socioeconomic status had very little effect on ethnic differences in estimated VO_2 max_ (data available from authors).

## Discussion

This study provides evidence of lower levels of physical fitness in British South Asian children compared with white Europeans; black African–Caribbean children in contrast had higher levels of physical fitness compared to white Europeans. Lower levels of physical fitness were observed in girls compared to boys; this gender difference was consistent across all ethnic groups studied. The lower level of physical fitness in South Asians compared to white Europeans was at least partly explained by the lower levels of physical activity in South Asian children. The higher level of physical fitness in black African–Caribbeans compared to white Europeans was at least partly explained by the higher levels of physical activity and lower levels of adiposity in black African–Caribbean children. The lower levels of physical fitness observed in girls could be partly (though not completely) explained by the lower physical activity levels in girls. Differences in adiposity did not contribute appreciably to South Asian–white European or gender differences in physical fitness.

### Relation to previous studies

Levels of estimated VO_2 max_ in boys and girls in the present study were slightly lower than those in children aged 10–12 years in a recent small UK-based study^25^ and were appreciably lower than those in children aged 9 years in the European Youth Heart Study,^26^ although the method of measuring VO_2 max_ differed between studies; physical activity levels in the present study were also markedly lower than in the European Youth Heart Study.^6^ ^10^ In the present study, girls had appreciably lower levels of physical fitness compared to boys, this finding is consistent with other studies in children of a similar age group in the UK^11^ ^25^ and Europe.^10^ The finding that South Asian boys and girls had lower levels of physical fitness compared to white European children is consistent with previous findings in UK adults^27^ ^28^ and with a small UK-based study of children aged 9 years;^11^ that study also reported that levels of physical fitness in black African–Caribbeans were similar to those of white European children, consistent with our findings.^11^ The finding that physical fitness was strongly related to physical activity in the present study is consistent with findings from other studies,^10^ ^25^ though no previous study has reported the contribution of physical activity to ethnic differences in physical fitness.

### Strengths and limitations

The submaximal fitness test used in the present study to predict VO_2 max_ has previously been used in a nationally representative sample of the English population^20^ and has been shown to capture two-thirds of the between-individual variance in the submaximal HR-VO_2_ relationship established using a wider intensity range during a more well-controlled stress-testing protocol (treadmill walking and running).^19^ Average levels and gender differences in estimated VO_2 max_ were consistent with those in UK children of a similar age group who underwent maximal fitness testing.^25^ The cross-sectional design of this study means that we cannot infer that the association we observed between physical fitness and physical activity is causal; however, evidence from randomised controlled trials has shown that interventions to increase activity levels in children and adolescents improve physical fitness,^29–31^ providing support for this direction of causality. The study included balanced representation of South Asians, black African–Caribbeans and white Europeans with ethnic comparisons carried out on a within-school basis to limit confounding. Further strengths of this study include the objective and validated measurement of physical activity levels using accelerometry^32^ and the measurement of adiposity using bioelectrical impedance with ethnic- and gender-specific equations for the prediction of fat mass, a method which has been shown to give more valid assessment of adiposity in a multi-ethnic population.^16^ Although the response rate in the study was modest (63%) and estimated VO_2 max_ was obtained for only 75% of the study sample who completed the step test, the socio-demographic and anthropometric characteristics of those participants were similar in those with and without physical fitness data, suggesting that the observed ethnic differences in physical fitness are not likely to be biased.

### Implications

The results presented in this study showed that South Asians had lower levels and black African–Caribbeans had higher levels of physical fitness compared to white European children, and that these ethnic differences were at least partly explained by lower levels of physical activity among South Asian children and by higher levels of physical activity and lower levels of adiposity among black African–Caribbean children. However, ethnic differences in physical activity did not appear to provide a complete explanation for these differences in physical fitness. Lower levels of physical fitness are associated with higher levels of adiposity, insulin resistance and cardiovascular risk.^7^ ^10^ ^25^ South Asian children have higher levels of adiposity, insulin resistance and hyperglycaemia, and black African–Caribbean children and adolescents have higher levels of insulin resistance and hyperglycaemia compared to white Europeans.^4^ The lower levels of physical fitness among South Asians observed the present study (by ∼2%) could help to explain the early development of type 2 diabetes risk among South Asians; this will be an important area for further investigation. In contrast, the higher levels of fitness observed in black African–Caribbean children do not help to explain increased risks of type 2 diabetes in this group. Improvements in physical fitness (potentially through increases in physical activity) could be important for chronic disease prevention in the UK South Asian population.

## References

[1] Health Survey for England 2004: The health of ethnic minority groups. In: Sproston K, Mindell J, eds. The NHS Information Centre, 2006. http://www.hscic.gov.uk/catalogue/PUB01209/heal-surv-hea-eth-min-hea-tab-eng-2004-rep.pdf

[2] Wild SH, Fischbacher C, Brock A Mortality from all causes and circulatory disease by country of birth in England and Wales 2001–2003. J Public Health (Oxf) 2007;29:191–8. 10.1093/pubmed/fdm010 17456532

[3] Whincup PH, Gilg JA, Papacosta O Early evidence of ethnic differences in cardiovascular risk: cross sectional comparison of British South Asian and white children. BMJ 2002;324:635 10.1136/bmj.324.7338.635 11895820 PMC84394

[4] Whincup PH, Nightingale CM, Owen CG Early emergence of ethnic differences in type 2 diabetes precursors in the UK: the Child Heart and Health Study in England (CHASE Study). PLoS Med 2010;7:e1000263 10.1371/journal.pmed.1000263 20421924 PMC2857652

[5] Fischbacher CM, Hunt S, Alexander L How physically active are South Asians in the United Kingdom? A literature review. J Public Health (Oxf) 2004;26:250–8. 10.1093/pubmed/fdh158 15454592

[6] Owen CG, Nightingale CM, Rudnicka AR Ethnic and gender differences in physical activity levels among 9-10-year-old children of white European, South Asian and African-Caribbean origin: the Child Heart Health Study in England (CHASE Study). Int J Epidemiol 2009;38:1082–93. 10.1093/ije/dyp176 19377098 PMC2720395

[7] Carnethon MR, Gidding SS, Nehgme R Cardiorespiratory fitness in young adulthood and the development of cardiovascular disease risk factors. JAMA 2003;290:3092–100. 10.1001/jama.290.23.3092 14679272

[8] LaMonte MJ, Eisenman PA, Adams TD Cardiorespiratory fitness and coronary heart disease risk factors: the LDS Hospital Fitness Institute cohort. Circulation 2000;102:1623–8. 10.1161/01.CIR.102.14.1623 11015338

[9] LaMonte MJ, Barlow CE, Jurca R Cardiorespiratory fitness is inversely associated with the incidence of metabolic syndrome: a prospective study of men and women. Circulation 2005;112:505–12. 10.1161/CIRCULATIONAHA.104.503805 16009797

[10] Ekelund U, Anderssen SA, Froberg K Independent associations of physical activity and cardiorespiratory fitness with metabolic risk factors in children: the European youth heart study. Diabetologia 2007;50:1832–40. 10.1007/s00125-007-0762-5 17641870

[11] Bettiol H, Rona RJ, Chinn S Variation in physical fitness between ethnic groups in nine year olds. Int J Epidemiol 1999;28:281–6. 10.1093/ije/28.2.281 10342692

[12] Williams PT Physical fitness and activity as separate heart disease risk factors: a meta-analysis. Med Sci Sports Exerc 2001;33:754–61. 10.1097/00005768-200105000-00012 11323544 PMC2821586

[13] Myers J, Kaykha A, George S Fitness versus physical activity patterns in predicting mortality in men. Am J Med 2004;117:912–18. 10.1016/j.amjmed.2004.06.047 15629729

[14] Rizzo NS, Ruiz JR, Hurtig-Wennlöf A Relationship of physical activity, fitness, and fatness with clustered metabolic risk in children and adolescents: the European youth heart study. J Pediatr 2007;150:388–94. 10.1016/j.jpeds.2006.12.039 17382116

[15] Donin AS, Nightingale CM, Owen CG Ethnic differences in blood lipids and dietary intake between UK children of black African, black Caribbean, South Asian, and white European origin: the Child Heart and Health Study in England (CHASE). Am J Clin Nutr 2010;92:776–83. 10.3945/ajcn.2010.29533 20739425 PMC7612313

[16] Nightingale CM, Rudnicka AR, Owen CG Are ethnic and gender specific equations needed to derive fat free mass from bioelectrical impedance in children of South Asian, black African-Caribbean and white European origin? Results of the Assessment of Body Composition in Children Study. PLoS ONE 2013;8:e76426 10.1371/journal.pone.0076426 24204625 PMC3799736

[17] Nightingale CM, Rudnicka AR, Owen CG Patterns of body size and adiposity among UK children of South Asian, black African-Caribbean and White European origin: Child Heart And health Study in England (CHASE Study). Int J Epidemiol 2011;40:33–44. 10.1093/ije/dyq180 21044977 PMC3043281

[18] Brage S, Brage N, Franks PW Reliability and validity of the combined heart rate and movement sensor Actiheart. Eur J Clin Nutr 2005;59:561–70. 10.1038/sj.ejcn.1602118 15714212

[19] Brage S, Ekelund U, Brage N Hierarchy of individual calibration levels for heart rate and accelerometry to measure physical activity. J Appl Physiol 2007;103:682–92. 10.1152/japplphysiol.00092.2006 17463305

[20] Health Survey for England (2008). http://www.hscic.gov.uk/catalogue/PUB00430/heal-surv-phys-acti-fitn-eng-2008-rep-v2.pdf.

[21] Tanaka H, Monahan KD, Seals DR Age-predicted maximal heart rate revisited. J Am Coll Cardiol 2001;37:153–6. 10.1016/S0735-1097(00)01054-8 11153730

[22] Henry CJ Basal metabolic rate studies in humans: measurement and development of new equations. Public Health Nutr 2005;8:1133–52. 10.1079/PHN2005801 16277825

[23] Thomas C, Nightingale CM, Donin AS Socio-economic position and type 2 diabetes risk factors: patterns in UK children of South Asian, black African-Caribbean and white European origin. PLoS ONE 2012;7:e32619 10.1371/journal.pone.0032619 22412897 PMC3296720

[24] Owen CG, Nightingale CM, Rudnicka AR Physical activity, obesity and cardiometabolic risk factors in 9- to 10-year-old UK children of white European, South Asian and black African-Caribbean origin: the Child Heart And health Study in England (CHASE). Diabetologia 2010;53:1620–30. 10.1007/s00125-010-1781-1 20454952 PMC2892063

[25] Boddy LM, Murphy MH, Cunningham C Physical activity, cardiorespiratory fitness, and clustered cardiometabolic risk in 10- to 12-year-old school children: the REACH Y6 study. Am J Hum Biol 2014;26:446–51. 10.1002/ajhb.22537 24599609

[26] Adegboye AR, Anderssen SA, Froberg K Recommended aerobic fitness level for metabolic health in children and adolescents: a study of diagnostic accuracy. Br J Sports Med 2011;45:722–8. 10.1136/bjsm.2009.068346 20558527

[27] Ghouri N, Purves D, McConnachie A Lower cardiorespiratory fitness contributes to increased insulin resistance and fasting glycaemia in middle-aged South Asian compared with European men living in the UK. Diabetologia 2013;56:2238–49. 10.1007/s00125-013-2969-y 23811809 PMC3764328

[28] Hall LM, Moran CN, Milne GR Fat oxidation, fitness and skeletal muscle expression of oxidative/lipid metabolism genes in South Asians: implications for insulin resistance? PLoS ONE 2010;5:e14197 10.1371/journal.pone.0014197 21152018 PMC2995737

[29] Annesi JJ, Westcott WL, Faigenbaum AD Effects of a 12-week physical activity protocol delivered by YMCA after-school counselors (Youth Fit for Life) on fitness and self-efficacy changes in 5-12-year-old boys and girls. Res Q Exerc Sport 2005;76:468–76. 10.1080/02701367.2005.10599320 16739685

[30] Faigenbaum AD, Westcott WL, Loud RL The effects of different resistance training protocols on muscular strength and endurance development in children. Pediatrics 1999;104:e5 10.1542/peds.104.1.e5 10390291

[31] Manios Y, Kafatos A, Mamalakis G The effects of a health education intervention initiated at first grade over a 3 year period: physical activity and fitness indices. Health Educ Res 1998;13:593–606. 10.1093/her/13.4.593 10345909

[32] Ekelund U, Sjostrom M, Yngve A Physical activity assessed by activity monitor and doubly labeled water in children. Med Sci Sports Exerc 2001;33:275–81. 10.1097/00005768-200102000-00017 11224818

