# Supplementary material for: Cross-sectional study of ethnic differences in physical fitness among children of South Asian, black African–Caribbean and white European origin: the Child Heart and Health Study in England (CHASE)
Source: BMJ Open. 2016 Jun 20;6(6):e011131. doi: 10.1136/bmjopen-2016-011131 (PMC4916632; doi:10.1136/bmjopen-2016-011131)
Supplement: Supplementary data [file bmjopen-2016-011131supp.pdf]

Supplementary material

Supplementary Figure 1: Distribution of estimated $VO_{2\ max}$

Supplementary Table 1: Distribution of estimated $VO_{2\ max}$ (ml O_2/min/kg) by sex and ethnic group

	All children			Boys			Girls		
Ethnic group or sub-group	n	Mean	SD	N	Mean	SD	N	Mean	SD
All children	1625	39.4	4.5	825	40.9	4.4	800	37.8	4.0
White European	424	39.6	4.6	231	40.7	4.6	193	38.2	4.2
South Asian	407	38.5	4.4	200	39.9	4.3	207	37.1	4.1
Indian	111	37.6	4.8	64	39.1	4.9	47	35.7	4.0
Pakistani	147	38.6	3.9	77	39.9	3.7	70	37.1	3.6
Bangladeshi	121	39.1	4.4	49	41.0	4.3	72	37.8	4.0
Black African-Caribbean	413	40.1	4.4	208	41.8	4.1	205	38.3	3.9
Black African	230	40.4	4.5	113	42.4	4.1	117	38.5	4.1
Black Caribbean	148	39.7	4.1	76	41.0	4.1	72	38.3	3.8
Other	381	39.3	4.5	186	41.0	4.4	195	37.7	3.9

South Asian other and black other subgroups are not included in the table therefore the numbers in the subgroups do not add up to the main ethnic group totals for South Asians and black African-Caribbeans

Supplementary Table 2: Ethnic differences in physical activity and adiposity

Outcome	Mean/geometric mean* (95% CI), p-value for difference from white Europeans										
	White European (n = 324)		South Asian (n =278)			Black African-Caribbean (n = 320)			Other (n = 293)		
Counts	401,758	(392,024, 411,491)	381,981	(370,884, 393,078)	0.002	414,900	(405,040, 424,761)	0.02	394,651	(384,606, 404,696)	0.22
CPM	501	(488, 513)	459	(445, 473)	<0.0001	500	(488, 513)	0.96	478	(465, 491)	0.002
Steps	10,356	(10,144, 10,567)	9,550	(9,311, 9,788)	<0.0001	9,928	(9,714, 10,142)	<0.001	10,000	(9,782, 10,217)	0.002
MVPA (min)	71	(68, 74)	65	(62, 68)	<0.0001	72	(69, 75)	0.15	69	(66, 71)	0.05
FMI (kg/m^5)*	2.10	(2.01, 2.18)	2.19	(2.09, 2.29)	0.17	1.90	(1.83, 1.98)	<0.001	2.18	(2.09, 2.27)	0.20

All means are adjusted for sex, age quartiles, month and school (random effect)
Abbreviations: CPM, counts per minute; FMI, fat mass index; MVPA, moderate to vigorous physical activity

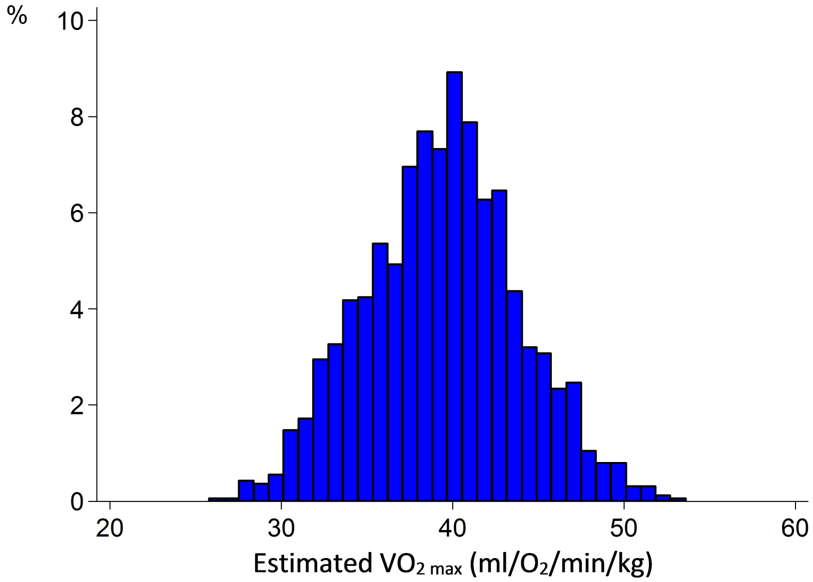

Supplementary Figure 1: Distribution of estimated VO2 max
